# Stable Anatomy Detection in Multimodal Imaging Through Sparse Group Regularization: A Comparative Study of Iron Accumulation in the Aging Brain

**DOI:** 10.3389/fnhum.2021.641616

**Published:** 2021-02-23

**Authors:** Matthew Pietrosanu, Li Zhang, Peter Seres, Ahmed Elkady, Alan H. Wilman, Linglong Kong, Dana Cobzas

**Affiliations:** ^1^Department of Mathematical and Statistical Sciences, University of Alberta, Edmonton, AB, Canada; ^2^Department of Biomedical Engineering, University of Alberta, Edmonton, AB, Canada; ^3^Department of Biomedical Engineering, McGill University, Montreal, QC, Canada; ^4^Department of Computing Science, University of Alberta, Edmonton, AB, Canada; ^5^Department of Computer Science, MacEwan University, Edmonton, AB, Canada

**Keywords:** ADMM, geometric regularization, group lasso, joint region, lasso, multiple sclerosis, sparse detection, total variation

## Abstract

Multimodal neuroimaging provides a rich source of data for identifying brain regions associated with disease progression and aging. However, present studies still typically analyze modalities separately or aggregate voxel-wise measurements and analyses to the structural level, thus reducing statistical power. As a central example, previous works have used two quantitative MRI parameters—R2* and quantitative susceptibility (QS)—to study changes in iron associated with aging in healthy and multiple sclerosis subjects, but failed to simultaneously account for both. In this article, we propose a unified framework that combines information from multiple imaging modalities and regularizes estimates for increased interpretability, generalizability, and stability. Our work focuses on joint region detection problems where overlap between effect supports across modalities is encouraged but not strictly enforced. To achieve this, we combine *L*_1_ (lasso), total variation (TV), and *L*_2_ group lasso penalties. While the TV penalty encourages geometric regularization by controlling estimate variability and support boundary geometry, the group lasso penalty accounts for similarities in the support between imaging modalities. We address the computational difficulty in this regularization scheme with an alternating direction method of multipliers (ADMM) optimizer. In a neuroimaging application, we compare our method against independent sparse and joint sparse models using a dataset of R2* and QS maps derived from MRI scans of 113 healthy controls: our method produces clinically-interpretable regions where specific iron changes are associated with healthy aging. Together with results across multiple simulation studies, we conclude that our approach identifies regions that are more strongly associated with the variable of interest (e.g., age), more accurate, and more stable with respect to training data variability. This work makes progress toward a stable and interpretable multimodal imaging analysis framework for studying disease-related changes in brain structure and can be extended for classification and disease prediction tasks.

## 1. Introduction

Following progressive developments in healthcare, the subpopulation of individuals over 60 years old has become the fastest growing worldwide (lr1, 2015). Thanks also in part to rapid advancements in neuroimaging and data storage technology, it is not surprising that much work in recent years has been devoted to studying structural changes in the brain during natural aging and disease progression (e.g., Alzheimer's and Parkinson's diseases Wyss-Coray, 2016 and multiple sclerosis Elkady et al., 2017) as well as differences between healthy and non-normative subjects, particularly in the elderly population.

The naturally high dimensionality of neuroimaging data and their inherent spatial correlation structures present significant challenges to the above goals. Implications of these challenges are apparent in the drawbacks (Davatzikos, 2004) of voxel-based analyses (Ashburner and Friston, 2000) and motivate many of the dimension reduction and regularization techniques commonly applied to neuroimaging data. These methods include pattern identification and discrimination (Fan et al., 2007), which rely on sample sizes not feasible in small neuroimaging studies, and sparse regularization for feature extraction or selection (Batmanghelich et al., 2011; Sabuncu and Van Leemput, 2012; He et al., 2016; Yu et al., 2019; Su et al., 2020). Despite successful applications of the latter to medical data (Krishnapuram et al., 2005; Zou and Hastie, 2005; Ryali et al., 2010), these approaches are unstable with respect to selected or extracted features. In the presence of spatial correlation structures, such as in neuroimaging data where each voxel carries similar information to its neighbors, sparsity comes at the severe detriment of interpretability. To address this, approaches to shape regularization have been developed, notably, total variation (TV) penalties which penalize spatial gradients to encourage a model to apply similar weights to neighboring voxels.

Nonetheless, incorporating multimodal imaging data into analytic techniques remains a difficult problem in general. Previous neuroimaging studies have demonstrated that the simultaneous analysis of multiple imaging modalities can improve statistical power (Elkady et al., 2017), although methods for efficiently doing so are limited. As a result, most studies either analyze different imaging modes independently (Betts et al., 2016) or aggregate voxel-wise observations to the structural level (Cherubini et al., 2009). This approach is inefficient from the perspective of both statistical power and estimate interpretability.

As a focal example and application for this article, we consider specific iron changes in deep gray matter (DGM), which have been histologically associated with healthy aging in the brain (Hallgren and Sourander, 1958). Gradient-echo magnetic resonance imaging (MRI) is used extensively *in vivo* due to its sensitivity to such changes (Peters, 2002). Transverse relaxation rate (R2*) and quantitative susceptibility (QS), both voxel-wise measures derived from gradient-echo MRI, are typically used for this purpose (Haacke et al., 2010; Acosta-Cabronero et al., 2016). Several studies have demonstrated a positive association between iron levels and age in healthy controls using quantitative MRI methods, although they consider only a single MRI measure or anatomical structure (Cherubini et al., 2009; Haacke et al., 2010; Acosta-Cabronero et al., 2016). The combined use of R2* and QS has only recently been introduced, for the purpose of improving statistical power in delineating iron and non-iron changes in studies of multiple sclerosis (Elkady et al., 2017). This earlier study, however, only combines modalities in an initial feature selection stage rather than in a single model.

Related works have similarly investigated, with the previously-discussed methodological limitations, the complex associations between QS and R2* measures, iron, and myelin in brain structures. Through a biochemical and histological analysis of postmortem brains using Pearson correlation (e.g., between myelin and R2*) and linear mixed effect regression (e.g., of R2* on iron and myelin intensities) on region- or structurally-aggregated measures, Hametner et al. (2018) showed that R2* is only weakly sensitive to myelin in white matter regions that do not contain iron, but is strongly sensitive to iron in the basal ganglia and white matter regions that do contain iron. The authors further demonstrated that QS also has mixed sensitivities to iron and myelin in white matter. Taege et al. (2019) applied region of interest-based (i.e., structurally-aggregated) regression analyses using R2* and QS to investigate deep gray matter microstructure in the context of multiple sclerosis. Mangeat et al. (2015) used two-dimensional independent components analysis of magnetization transfer, cortical thickness, *B*_0_ orientation, and R2* to extract information about the fraction of myelin in white matter. Bergsland et al. (2018) used QS and diffusion metrics to perform separate univariate statistical analyses of thalamic white matter tracts and used non-parametric correlation analysis to combine these measures and quantify their association with clinical disease metrics.

While all the previous works provide useful insight into the structure and composition of the brain, the use of region-based analyses relies of the typically incorrect assumption of intra-region homogeneity. Furthermore, these works generally do not jointly incorporate multiple imaging modes as model covariates. These are general limitations in the application of traditional statistical methods to high-dimensional neuroimaging data. In this work, we aim to address these limitations in the context of region-detection problems.

In this article, we propose a unified, multimodal framework for joint region detection associated with a numerical variable of interest such as age. We formulate this problem in terms of sparse regression with *L*_1_ (lasso), TV, and *L*_2_ group lasso penalties, with the last of these combining information across multiple modalities. These penalties have been widely used in the development of robust methods for medical data (He et al., 2016; Yu et al., 2019). Together, the penalties regularize spatial effect estimates for better interpretability, both within and between modalities. This work is concerned primarily with joint region detection, such as in our focal R2*-QS-age problem, where overlap between effect supports across modalities is encouraged but not strictly enforced. To address the computational difficulty in implementing these penalties together, we propose an optimization procedure using the alternating direction method of multipliers (ADMM) algorithm.

Our work addresses the need for multimodal neuroimaging techniques for joint region detection that can provide interpretable estimates accounting for similarities in spatial effect supports. In our focal example, it is critical to account for both R2* and QS since, voxel-wise, both are positively associated with iron accumulation (Langkammer et al., 2012): the increased statistical power in multimodal MRI analysis has previously detected subtle pathological changes in neurological diseases such as multiple sclerosis (Elkady et al., 2017) and will similarly improve the detection of aging-related neurodegenerative effects. Generally, our approach emphasizes the interpretability and stability of support estimates in terms of spatial smoothness and variability (with respect to perturbations in training data), respectively. Our estimators are much less affected by spatial correlation structures that would otherwise result in severe undersegmentation in other sparse analyses. Furthermore, our work easily accommodates multiple anatomical regions simultaneously: this contrasts with traditional voxel-based analyses, which are generally known to have unsuitable performance when considering multiple regions due to their disregard for spatial correlation (Ashburner and Friston, 2000). In the limited literature addressing multimodal data (Michel et al., 2011; Gramfort et al., 2013), our formulation using sparse regularization is novel.

## 2. Materials and Methods

### 2.1. Sparse Regression and Image Regularization

We first present more detail on the components of our proposed framework and define the two other models against which the proposed method will be compared. For notational simplicity, we ignore intercept terms in the following subsections and only consider two modalities, although our setup generalizes immediately.

Let *X*_1_ and *X*_2_ be *n* × *p* matrices corresponding to the two imaging modalities (e.g., R2* and QS) after columnwise standardization. These matrices have *i*-th row $x_{1i}^{⊤}$ and $x_{2i}^{⊤}$, respectively, corresponding to vectorized, observed image data for the *i*-th subject. In general, *p* can be smaller than the number of voxels in the image due to the application of a mask (that is constant across subjects). Let ***β***_1_ and ***β***_2_ denote corresponding voxel effects that are to be estimated, and ***y*** an observed length-*n* vector of a continuous variable corresponding to the *n* subjects. Loosely, we are interested in associations of *X*_1_ and *X*_2_ with ***y***, where *p* ≫ *n*.

#### 2.1.1. Sparse Regression

The traditional (least squares) linear regression problem in this setting takes the form $\left({\hat{\beta }}_{1},{\hat{\beta }}_{2}\right)=\arg\underset{\beta_{1},\beta_{2}}{\min}\|y-X_{1}\beta_{1}-X_{2}\beta_{2}{\|}_{2}^{2}$. This estimator is unstable and has extremely high variability, attributable to overfitting in this high-dimensional setting. Furthermore, this estimator typically yields estimates with only non-zero entries, making it useless for region identification. To address both these problems, we introduce a sparsity assumption on $\beta ={\left(\beta_{1}^{⊤},\beta_{2}^{⊤}\right)}^{⊤}$ by adding an *L*_1_ (lasso) penalty to the previous problem, as an approximation of the *L*_0_ penalty used in (NP-hard) best subset selection (Tibshirani, 1996; Huo and Ni, 2007). The lasso penalty is widely-established in both statistical theory and application as a method for variable selection (Tibshirani, 1996; Tibshirani et al., 2005; Simon et al., 2013): loosely-speaking, when applied in 3D imaging contexts, this penalty identifies voxels of interest by shrinking entries of $\hat{\beta }$ corresponding to “non-significant” voxels to zero.

The sparse regression problem becomes

(1) $$\begin{array}{l} \underset{\beta_{1},\beta_{2}}{\arg\min}\;\|y-X_{1}\beta_{1}-X_{2}\beta_{2}{\|}_{2}^{2}+\lambda_{1}\|\beta_{1}{\|}_{1}+\lambda_{1}\|\beta_{2}{\|}_{1}, \end{array}$$

where the hyperparameter λ_1_ controls the balance between model fidelity and model sparsity. We use the same hyperparameter λ_1_ for both ***β***_1_ and ***β***_2_ since *X*_1_ and *X*_2_ are standardized.

While the sparse regression problem in (1) is typically encountered in models for predicting *y*, that is not our primary interest here. We are instead concerned with associations of *X*_1_ and *X*_2_ with ***y***: in a neuroimaging context, we wish to identify regions with MRI measures *X* that vary together with age ***y***. In particular, the unpenalized, least squares model maximizes the square sample correlation between ***y***_*i*_ and $x_{1i}^{⊤}\beta_{1}+x_{2i}^{⊤}\beta_{2}$, but requires further regularization to become practically usable.

#### 2.1.2. TV Regularization

While the previous sparsity assumption solves initial problems in estimating ***β***, it does not suitably address estimate interpretability. In neuroimaging contexts, since measures from nearby voxels tend to be correlated, $\hat{\beta }$ will select isolated voxels rather than contiguous, compact regions that are more amenable to interpretation (Kandel et al., 2013; Dubois et al., 2014; Eickenberg et al., 2015).

To encourage interpretability, we add an image-based penalty to (1). Both TV (Michel et al., 2011; Baldassarre et al., 2012; Gramfort et al., 2013; Dohmatob et al., 2014; Dubois et al., 2014; Eickenberg et al., 2015) and GraphNet (Ng et al., 2010; Grosenick et al., 2013; Kandel et al., 2013; Watanabe et al., 2014) penalties have been explored in sparse regression and classification contexts: we use the former as it has demonstrated superior performance with respect to variable selection (Michel et al., 2011; Gramfort et al., 2013; Eickenberg et al., 2015). The anisotropic TV penalty (Tibshirani et al., 2005; Watanabe et al., 2014) is an *L*_1_ penalty on the gradient of an image, which we denote by

$$\|\nabla \beta {\|}_{1}=\|\nabla_{1}\beta {\|}_{1}+\|\nabla_{2}\beta {\|}_{1}+\|\nabla_{3}\beta {\|}_{1},$$

where ∇_*i*_ (for *i* = 1, 2, 3) denotes a gradient taken along the *i*-th orthogonal coordinate direction. The resulting penalized problem is

(2) $$\begin{array}{l} \underset{\beta_{1},\beta_{2}}{\arg\min}\;\|y-X_{1}\beta_{1}-X_{2}\beta_{2}{\|}_{2}^{2}+\lambda_{1}\|\beta_{1}{\|}_{1}+\lambda_{1}\|\beta_{2}{\|}_{1}\\ +\lambda_{2}\|\nabla \beta_{1}{\|}_{1}+\lambda_{2}\|\nabla \beta_{2}{\|}_{1}, \end{array}$$

where λ_2_ is another hyperparameter.

#### 2.1.3. Sparse Group Lasso Regularization

The TV penalty in (2) addresses the interpretability of ***β***_1_ and ***β***_2_ estimates separately rather than jointly. The latter is a natural consideration in neuroimaging when interested in the joint support, e.g., regions that are associated with age for both R2* and QS modalities, or if prior knowledge suggests that the effect supports should overlap. We introduce an additional sparse group lasso regularization term to this end.

Group lasso methods apply a penalty to pre-specified groups of variables and are widely used. To encourage overlap in the two estimate supports, we consider *p* groups of size 2, each composed of voxel-wise measures at the same location. The sparse group lasso penalty (Simon et al., 2013) in this case is given by

$$\lambda_{3}\overset{p}{\underset{j=1}{\sum }}\sqrt{\beta_{1j}^{2}+\beta_{2j}^{2}}.$$

In contrast, the standard group lasso (Tibshirani, 1996) applies an *L*_1_ penalty to the estimates in each group rather than the *L*_2_ penalty above, forcing all estimates in a group to be both either zero or non-zero. This is an undesireably hard constraint in our setting.

The resulting penalized problem and our proposed estimator is given by

(3) $$\begin{array}{l} \underset{\beta_{1},\beta_{2}}{\arg\min}\;\|y-X_{1}\beta_{1}-X_{2}\beta_{2}{\|}_{2}^{2}+\lambda_{1}\|\beta_{1}{\|}_{1}+\lambda_{1}\|\beta_{2}{\|}_{1}\\ +\lambda_{2}\|\nabla \beta_{1}{\|}_{1}+\lambda_{2}\|\nabla \beta_{2}{\|}_{1}+\lambda_{3}\overset{p}{\underset{j=1}{\sum }}\sqrt{\beta_{1j}^{2}+\beta_{2j}^{2}}. \end{array}$$

### 2.2. Optimization

The optimization problem in (3) is difficult to solve due to the non-differentiability of the *L*_1_ norm appearing in the lasso and TV penalties, to which standard gradient-based methods do not apply. Various numerical methods and have been explored for settings with these two penalties, including for logistic regression (Dohmatob et al., 2014).

In this work, we apply the ADMM algorithm (Boyd et al., 2011), an efficient convex optimization scheme amenable to parallel computing. Its computational benefits rely on splitting a given problem into two convex sub-problems. In the present case, denoting $\beta ={\left(\beta_{1}^{⊤},\beta_{2}^{⊤}\right)}^{⊤}$, (3) can be written as

(4) $$\begin{array}{l} \underset{\beta ,\alpha }{\arg\min}L\left(\beta \right)+\|\Lambda^{⊤}\alpha {\|}_{1}\\ \text{subject to }\beta =A\alpha , \end{array}$$

where $L\left(\beta \right)=\|y-X_{1}\beta_{1}-X_{2}\beta_{2}{\|}_{2}^{2}+\lambda_{3}\overset{p}{\underset{i=1}{\sum }}\sqrt{\beta_{1j}^{2}+\beta_{2j}^{2}}$ is a smooth function of ***β***, $A={\left[I_{p}|D^{⊤}\right]}^{⊤}$, $\Lambda ={\left[\lambda_{1}I_{p}|\lambda_{2}I_{3p}\right]}^{⊤}$, and *D* is the 3-dimensional differential operator (Rohr, 1997).

The ADMM algorithm solves (4) using the iterative updates

$$\begin{array}{l} \beta^{\left(t+1\right)}=\underset{\beta }{\arg\min}L_{\rho }\left(\beta ,\alpha^{\left(t\right)},\eta^{\left(t\right)}\right)\\ \alpha^{\left(t+1\right)}=\underset{\alpha }{\arg\min}L_{\rho }\left(\beta^{\left(t+1\right)},\alpha ,\eta^{\left(t\right)}\right)\\ \eta^{\left(t+1\right)}=\eta^{\left(t\right)}+\rho \left(\alpha^{\left(t+1\right)}-A\beta^{\left(t+1\right)}\right), \end{array}$$

where ***η*** is an auxiliary variable and $L_{\rho }\left(\beta ,\alpha ,\eta \right)=L\left(\beta \right)+\|\Lambda^{⊤}\alpha {\|}_{1}+\eta^{⊤}\left(\beta -A\alpha \right)+\frac{\rho }{2}\|\beta -A\alpha {\|}_{2}^{2}$ is the augmented Lagrangian with parameter ρ.

The ***β***-update step minimizes a smooth function (noting that ***α*** and ***η*** are held fixed) and can be performed using standard first-order methods. The ***α***-update step, on the other hand, features a non-smooth objective function containing both *L*_2_ and *L*_1_ penalties on ***α***. This update admits a closed-form solution using the soft thresholding operator *S*_λ_(*x*) = sgn(*x*)(|*x*| − λ), namely,

$$\alpha_{i}^{\left(t+1\right)}=S_{\Lambda_{ii}}\left({\left(A\beta^{\left(t+1\right)}-\eta^{\left(t\right)}\right)}_{i}\right).$$

For termination criterion, we follow the suggestion of Boyd et al. (2011) based on the problem's primal residuals $r_{\text{primal}}^{\left(t\right)}=\alpha^{\left(t\right)}-A\beta^{\left(t\right)}$ and dual residuals $r_{\text{dual}}^{\left(t\right)}=\rho A^{⊤}\left(\alpha^{\left(t\right)}-\alpha^{\left(t-1\right)}\right)$, namely,

$$\|r_{\text{primal}}^{\left(t\right)}{\|}_{2}\leq \epsilon_{\text{primal}}\;\text{and}\;\|r_{\text{dual}}^{\left(t\right)}{\|}_{2}\leq \epsilon_{\text{dual}},$$

for positive primal and dual tolerances ϵ_primal_ and ϵ_primal_, respectively.

### 2.3. Models and Hyperparameter Tuning

In all subsequent analyses, we compare performance between three models. The first is an independent sparse (IS) model, composed of one model for each imaging modality, each fit with lasso and TV regularization. For this model, “predictions” for ***y*** are taken as averages across the independent models. Second, we consider a joint sparse (JS) model, given in (2), including all imaging modalities together with lasso and TV penalties. Third, we consider our proposed sparse group lasso (SGL) method in (3) including all imaging modalities and lasso, TV, and sparse group lasso regularization.

Generally, three hyperparameters λ_1_, λ_2_, and λ_3_ require tuning. We reparameterize these in terms of λ, *r*_1,2_, and *r*_3_ as

$$\left(\lambda_{1},\;\lambda_{2},\;\lambda_{3}\right)=\lambda \left(r_{1,2}\left(1-r_{3}\right),\;\left(1-r_{1,2}\right)\left(1-r_{3}\right),\;r_{3}\right)$$

and use the Bayesian information criterion (BIC) (Fan and Tang, 2013), calculated on the full training dataset, as a performance criterion to choose optimal hyperparameter values. In all analyses, results using the generalized information criterion (GIC) (Fan and Tang, 2013) are similar.

In the following simulation studies, (λ, *r*_1,2_, *r*_3_) are tuned on a grid over [*e*^−9^, 0] × [0.1, 0.9] × [0, 0.9]. As further restrictions, the IS model enforces *r*_3_ = 0 and the SGL model restricts *r*_1,2_ = 0.05 and *r*_3_ = 0.6. Tuning in the subsequent neuroimaging analysis restricts the maximum value of λ to *e*^−1^, but is otherwise identical.

### 2.4. Data, Analyses, and Evaluation

#### 2.4.1. Synthetic Data and Simulation Study

Because ground truth for the support regions is not available in real-world imaging, we use synthetic data to more comprehensively investigate the performance of our proposed method. Our setup generalizes that in Zhang et al. (2018) by including a second imaging modality and inter-modality correlation. In all simulation studies, we use training datasets of size *n* = 100, which is comparable to the *n* = 113 used in our real-world MRI application.

**Figure 1:**
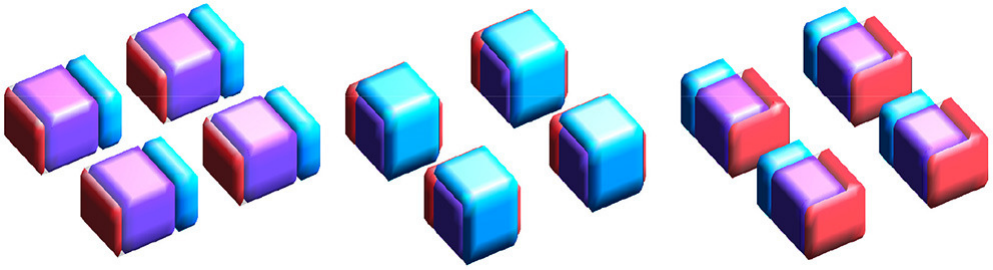
True supports for three of the 10 simulations considered. Purple represents the joint support, while red and cyan are support regions unique to exactly one imaging mode.

Imaging data for the first mode is generated with size 32×32×8. The true support is represented by four disjoint 8×8×4 regions (which we call “blocks”). Imaging data for the second mode is generated similarly: however, to simulate (partial) overlap in the support between the two modes, we allow block size for the second mode to vary uniform randomly over {6, 7, 8, 9, 10}^2^ × {4, 5}. Example supports are provided in Figure 1.

Background noise is independently generated from a N(0,I) distribution. Voxel measures within a block are correlated only with voxels from the same block and from the corresponding block in the other mode. This inter-voxel correlation is controlled by a spatial coherence parameter ρ that decreases with *L*_0_ distance, and is further multiplied by an inter-mode correlation parameter η for measures in different modes.

The true effect ***β*** is set to 0.1, 0.2, 0.3, or 0.4 in each block, for varying signal strength, and 0 elsewhere. The simulated response is generated as ***y*** = 0.01 + *X*_1_***β***_1_ + *X*_2_***β***_2_ + ε, where $\varepsilon \sim N\left(0,I_{n}\right)$, following the notation in section 2.

We consider two small-sample (*n* = 100, *p* = 32 × 32 × 8 × 2 = 16384) simulation settings: a “low correlation” (ρ = 0.5, η = 0.2) and a “high correlation” setting (ρ = 0.8, η = 0.5). In each case, we run two analyses. The first examines overall performance averaged over 10 simulations, each using independently-generated true supports. The second examines estimate stability over five independent datasets generated using the same true support (i.e., for 10 possible inter-simulation comparisons). From here on, we refer to these two analyses as simulation study #1 and simulation study #2, respectively.

Intuitively, simulation study #1 assesses average performance across a variety of ground truths. On the other hand, simulation study #2 assesses estimate stability across different training datasets for a single, fixed ground truth.

#### 2.4.2. Neuroimaging Data, Pre-processing, and Analyses

**Figure 2:**
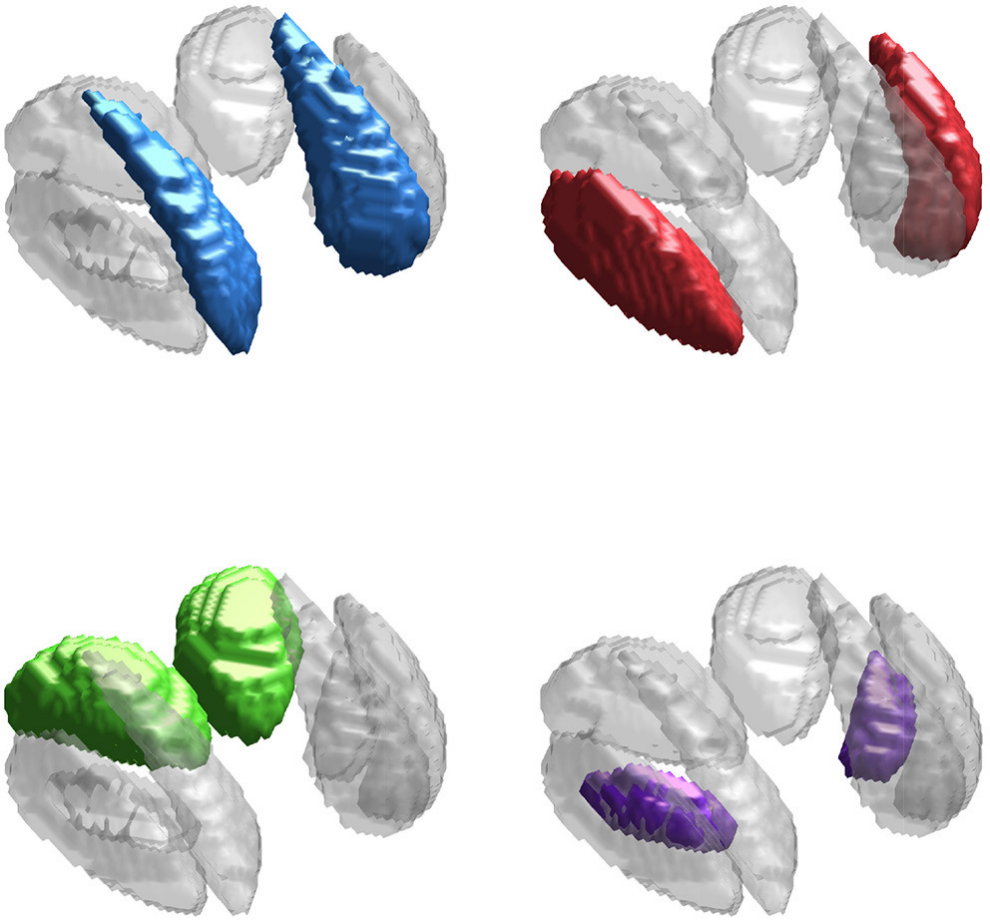
Deep gray matter (DGM) structures considered in this study. The DGM mask applied to the data is shown in gray in all sub-panels. The caudate (blue), putamen (red), thalamus (green), and globus pallidus (purple) are highlighted in each sub-panel.

The neuroimaging data used in this work is from an in-house study of multiple sclerosis and controls (Walsh et al., 2014; Elkady et al., 2017), similar to that used in Zhang et al. (2018) but composed of only *n* = 113 control scans (mean age 40.25 with SD 10.91, minimum 21.8, and maximum 65.1), of which 40 were obtained from male subjects. Our primary interest is in identifying regions of the brain in which variability in iron levels is associated with age. We focus on four subcortical deep gray matter (DGM) structures: the caudate, putamen, thalamus, and globus pallidus, shown in Figure 2 (Zhang et al., 2017, 2018). We use R2* and QS modalities, as both are known to be highly sensitive to changes in non-heme iron (Wang and Liu, 2015).

Imaging was conducted using a 4.7 T system (Varian Inova, Palo Alto, CA), with two imaging sequences obtained per session. R2* and QS maps were calculated using three-dimensional multi-echo gradient echo acquisitions (acquisition time = 9.4 min, repetition time 44 ms, number of echoes = 10, time to first echo = 2.93 ms, echo spacing = 4.1 ms, monopolar readout, flip angle = 10°, number of contiguous slices = 80, field of view = 160 × 256 × 160 mm^3^, voxel size 1 × 1 × 2 mm^3^). R2* maps were computed using a mono-exponential temporal fit of image magnitude, while QS maps were calculated using an image phase inversion (Bilgic et al., 2012) following phase unwrapping using FSL PRELUDE and background field removal using regularization-enabled sophisticated harmonic removal of phases (RESHARP) (Sun and Wilman, 2013). An anatomical MRI sequence was acquired by 3D T1w volumetric imaging using magnetization-prepared rapid gradient-echo (MPRAGE) (acquisition time = 4.8 min, flip angle = 108°, TE/TR = 4.5/8.5 ms, inversion time to start of readout = 300 ms, sequential phase encoding, number of contiguous slices = 84, voxel size = 0.9 × 0.9 × 2 mm^3^).

Prior to analysis, the MRI data was pre-processed and aligned with an in-house unbiased template using ANTs (ANTS, 2011), built from T1w, R2*, and QS data from 10 healthy controls. Pre-processing involved intra-subject alignment of the R2* and QS maps with the T1w map. Non-linear registration in the template space was accomplished by applying SyN (ANTS, 2011) to the multimodal MRI data. Observation row vectors $x_{i}^{⊤}$ were obtained by taking voxels within a DGM mask manually traced on the atlas, shown in **Figure 5**. Data matrix columns were standardized before analysis.

### 2.5. Evaluation Methodology

For simulation study #1, we report prediction mean absolute error (MAE) on the training set (*n* = 100) and both MAE and *R*^2^ on the independent testing set (*n* = 500). Since the true support is known for synthetic data, we also report Dice scores between the estimated and true supports (called “full Dice”) as well as between the estimated and true joint supports (called “joint Dice”). Here, “joint” refers to the region of overlap in the supports of the two imaging modes.

For simulation study #2, we additionally report Dice scores between estimated supports obtained from different training datasets. This performance measure quantifies the stability of the estimated support with respect to training set variability.

In the neuroimaging analysis, we consider a 23-fold cross-validation approach due to the small sample size. We report training and testing set MAE, testing set *R*^2^, and pairwise Dice scores between the supports estimated using each training dataset.

## 3. Results

### 3.1. Simulation Studies

**Figure 3:**
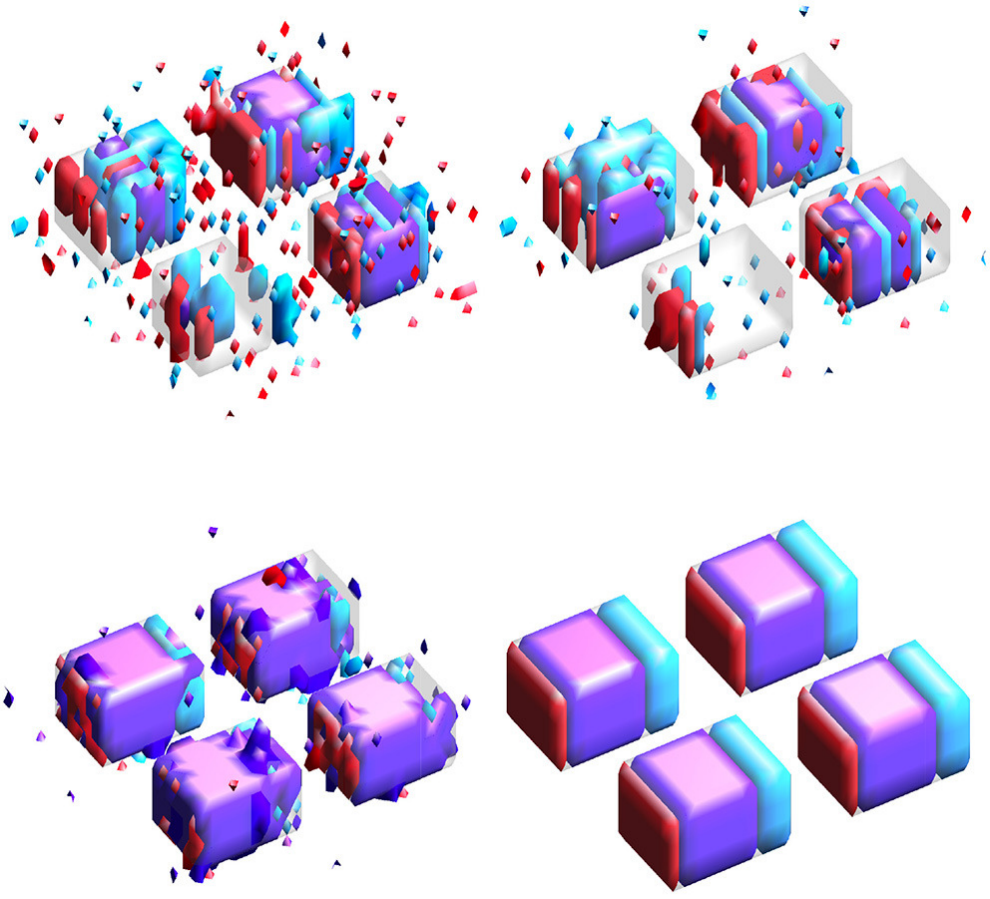
Estimated supports for one simulation in the HC setting. Purple represents the joint support, while red and cyan are support regions unique to exactly one imaging mode. In each sub-panel, the bottom, left, right, and top block corresponds to a true effect size of 0.1, 0.2, 0.3, and 0.4, respectively. **(Top-left)** Independent sparse (IS) model estimates. **(Top-right)** Joint sparse (JS) model estimates. **(Bottom-left)** Proposed sparse group lasso (SGL) model estimates. **(Bottom-right)** True support, also indicated in other sub-panels in gray, for reference.

Results for simulation study #1 are provided in Table 1. Figure 3 gives example visualizations of the estimated and true supports for one simulation. These results are similar between the high and low correlation settings. Full and joint Dice scores are significantly higher for the proposed SGL model, with 1.4–4.9 times lower variability across simulations, suggesting more accurate and stable support estimation when using the proposed SGL method. Figure 3 supports these conclusions and illustrates how the SGL estimator more clearly and accurately identifies the joint support: in contrast, IS and JS estimates suggest higher false-positive voxel selection rates. These points are visually evident though the differences in the size of the joint region (shown in purple) detected by the SGL and the IS/JS estimates, the noise present in the IS (and JS, to a lesser extent) estimate unique to one imaging modality (indicated in red and cyan), and the notable deterioration of IS/JS estimate quality when the true effect size is small. While not the primary goal, the proposed SGL model retains excellent association with the response, shown in testing set MAE and *R*^2^: as hypothesized, this suggests that the proposed SGL model generalizes better to new data and that the estimated relationship (in $\hat{\beta }$) between voxel-wise measures and the response is less-affected by over-fitting to the training dataset, relative to the IS and JS models.

**Table 1:** Performance results for simulation study #1, averaged over 10 independent simulations.

**Method**	**Setting**	**MAE**		**Test R^2^**	**Dice**	
		**Train**	**Test**		**Full**	**Joint**
**IS**	LC	0.187 (0.140)	29.286 (7.895)	0.732 (0.144)	0.427 (0.236)	0.552 (0.152)
	HC	0.147 (0.102)	27.840 (7.334)	0.762 (0.139)	0.532 (0.151)	0.596 (0.128)
**JS**	LC	**0.127 (0.056)**	29.060 (6.078)	0.734 (0.124)	0.380 (0.148)	0.537 (0.112)
	HC	**0.127 (0.056)**	29.066 (6.072)	0.734 (0.123)	0.380 (0.149)	0.537 (0.113)
**SGL**	LC	0.128 (0.055)	**8.310 (4.682)**	**0.998 (0.001)**	**0.641 (0.107)**	**0.824 (0.032)**
	HC	0.129 (0.055)	**8.309 (4.682)**	**0.998 (0.001)**	**0.641 (0.107)**	**0.823 (0.031)**

“*LC” and “HC” denote the low correlation (ρ = 0.5, η = 0.5) and high correlation (ρ = 0.8, η = 0.5) settings, respectively. “IS,” “JS,” and “SGL” refer to the independent sparse, joint sparse, and proposed sparse group lasso models, respectively. All results are presented in the form “mean (SD).” Entries with the best mean results for the LC and HC settings in each column are in bold*.

**Figure 4:**
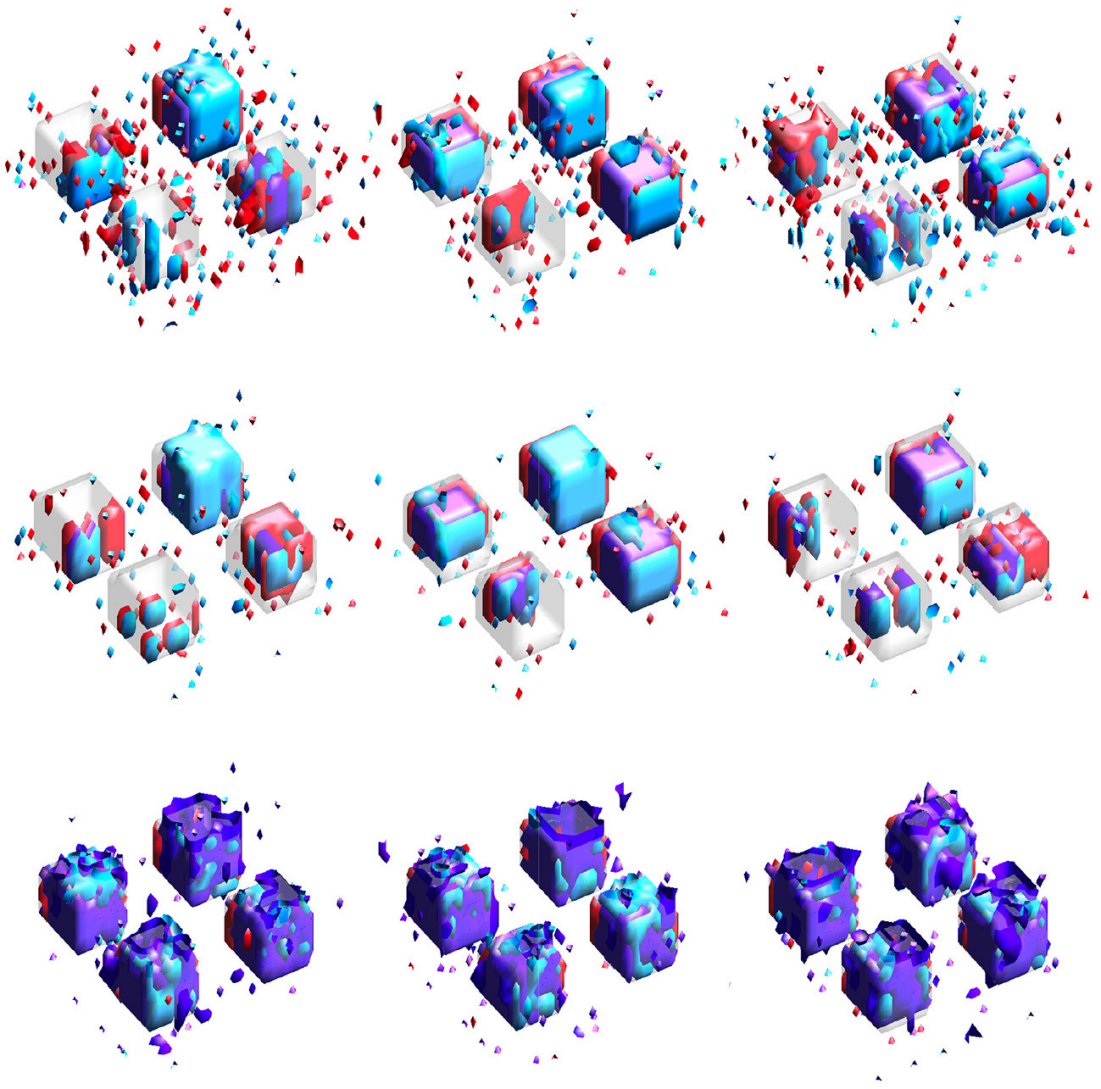
Estimated supports for different simulations with the same ground truth, shown in the middle panel of Figure 1. Purple represents the estimated joint support, while red and cyan are estimated support regions unique to exactly one imaging mode. The true support is indicated in gray, for reference. In each sub-panel, the bottom, left, right, and top block corresponds to a true effect size of 0.1, 0.2, 0.3, and 0.4, respectively. Estimates in the same column were obtained using the same dataset. **(Top)** Independent sparse (IS) model estimates. **(Middle)** Joint sparse (JS) model estimates. **(Bottom)** Proposed sparse group lasso (SGL) model estimates.

Results for simulation study #2 are provided in Table 2. Figure 4 gives example visualizations of the estimated supports (obtained from different training datasets) and the true support for one simulation. These results agree with the conclusions of simulation study #1: the proposed SGL method more accurately and stably selects the true support (either full or joint) while retaining high association with the response. Despite a more complex true support, Figure 4 demonstrates the stability of SGL estimates across independent training sets. This contrasts with IS and JS estimates, which again show higher false-positive voxel selection rates (visually, with red, cyan, or purple outside of the gray true support) and struggle to identify the joint support (visually, with estimates in purple), particularly when the true signal is weak.

**Table 2:** Performance results for simulation study #2.

**Method**	**Setting**	**MAE**		**Test R^2^**	**Dice (truth)**		**Dice (pairwise)**	
		**Train**	**Test**		**Full**	**Joint**	**Full**	**Joint**
**IS**	LC	0.207 (0.129)	25.940 (6.789)	0.811 (0.056)	0.641 (0.069)	0.659 (0.097)	0.579 (0.072)	0.688 (0.080)
	HC	0.207 (0.129)	25.940 (6.789)	0.811 (0.056)	0.641 (0.069)	0.659 (0.097)	0.579 (0.072)	0.688 (.080)
**JS**	LC	**0.112 (0.026)**	26.426 (6.902)	0.791 (0.067)	0.609 (0.110)	0.568 (0.190)	0.556 (0.076)	0.541 (0.153)
	HC	**0.112 (0.026)**	26.426 (6.902)	0.791 (0.067)	0.609 (0.110)	0.568 (0.190)	0.556 (0.076)	0.541 (0.153)
**SGL**	LC	0.144 (0.040)	**2.234 (0.402)**	**0.999 (0.001)**	**0.832 (0.012)**	**0.700 (0.024)**	**0.835 (0.007)**	**0.760 (0.011)**
	HC	0.141 (0.042)	**2.241 (0.404)**	**0.999 (0.001)**	**0.831 (0.011)**	**0.700 (0.021)**	**0.835 (0.006)**	**0.760 (0.009)**

“*LC” and “HC” denote the low correlation (ρ = 0.5, η = 0.5) and high correlation (ρ = 0.8, η = 0.5) settings, respectively. “IS,” “JS,” and “SGL” refer to the independent sparse, joint sparse, and proposed sparse group lasso models, respectively. All results are presented in the form “mean (SD).” Entries with the best mean results for the LC and HC settings in each column are in bold*.

### 3.2. Neuroimaging Data Analysis

**Figure 5:**
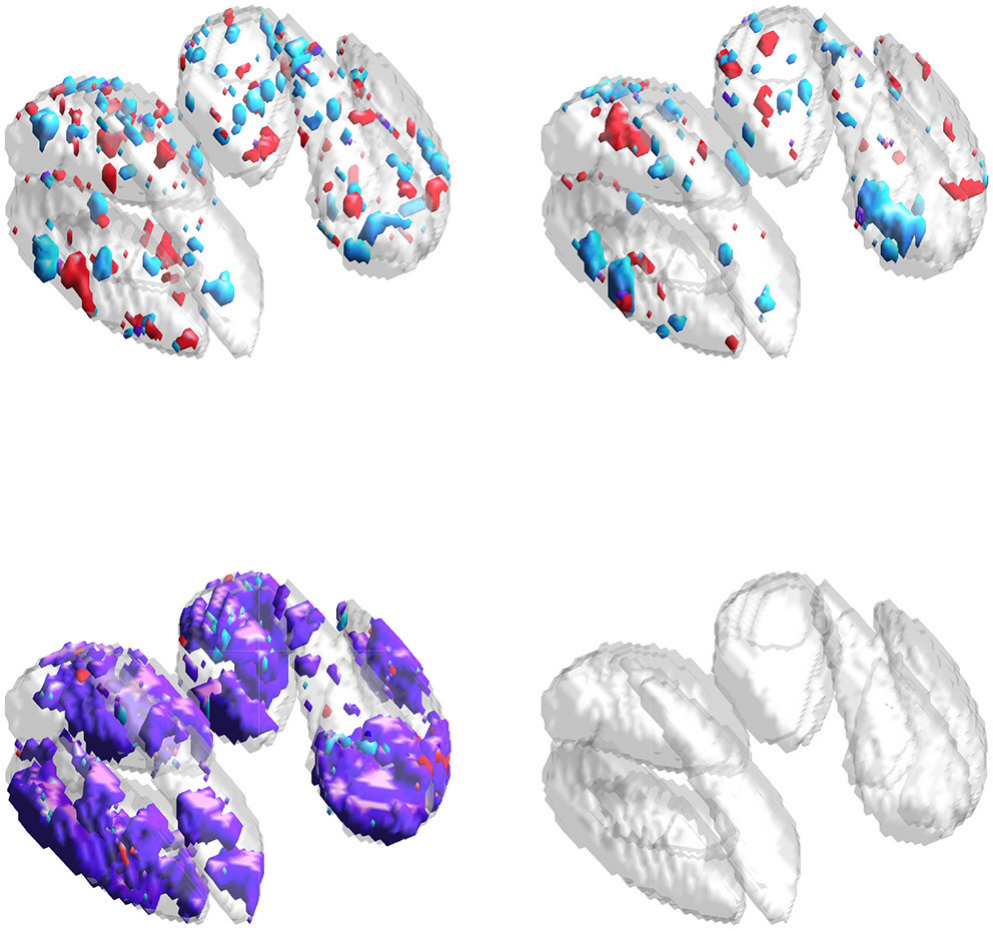
Estimates obtained from one training fold in the neuroimaging data analysis. Purple represents the estimated joint support, while red and cyan are estimated support regions unique to exactly one imaging modality. **(Top-left)** IS model estimates. **(Top-right)** JS model estimates. **(Bottom-left)** SGL model estimates. **(Bottom-right)** DGM mask, also included in other sub-panels for reference.

**Figure 6:**
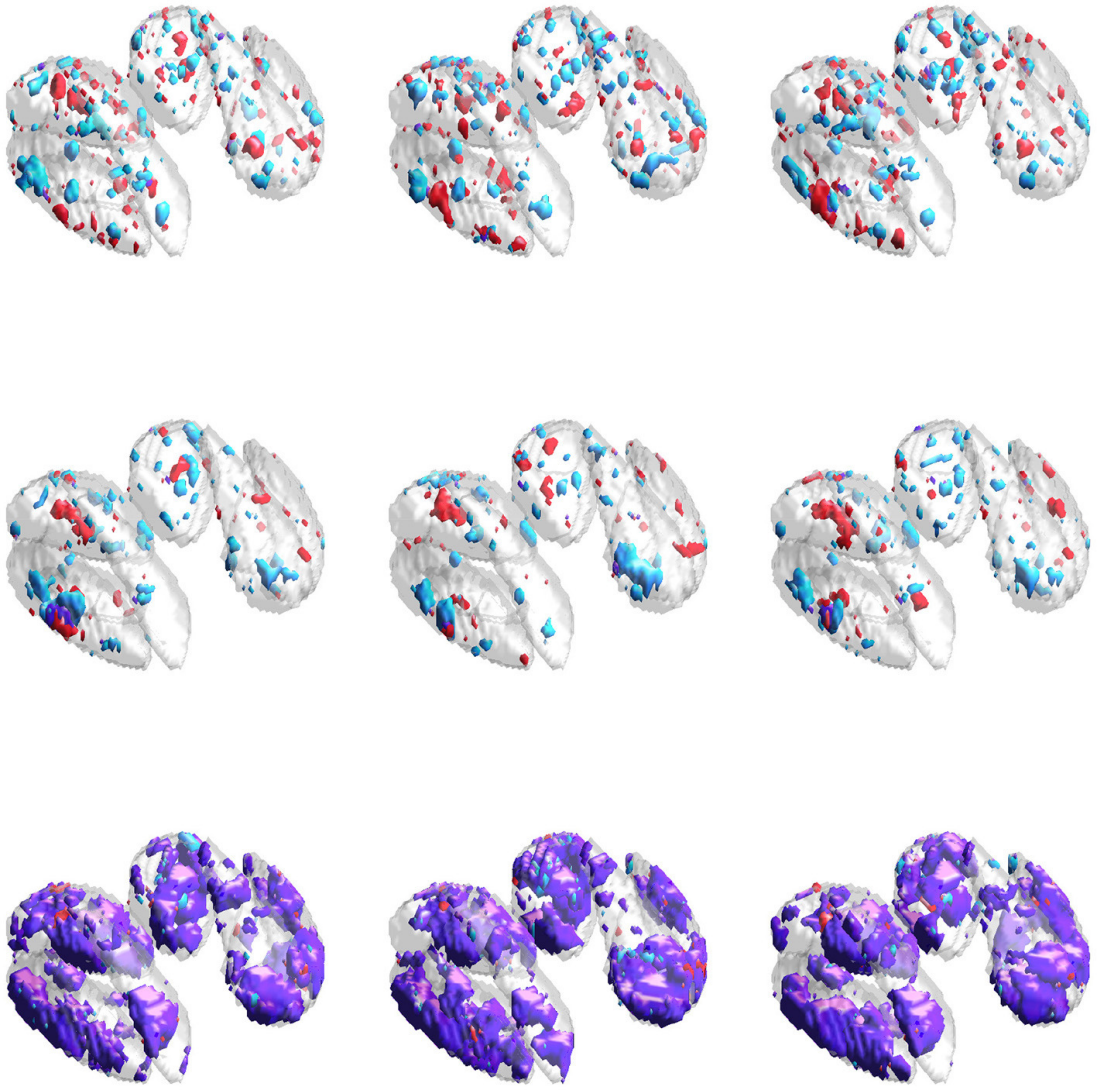
Estimated supports for different training datasets in the neuroimaging data analysis. Purple represents the estimated joint support, while red and cyan are estimated support regions unique to exactly one imaging modality. The DGM mask is shown in gray in all sub-figures, for reference. Estimates in the same column were obtained using the same dataset. **(Top)** Independent sparse (IS) model estimates. **(Middle)** Joint sparse (JS) model estimates. **(Bottom)** Proposed sparse group lasso (SGL) model estimates.

Results for the neuroimaging analysis are given in Table 3. Examples of the estimated supports are provided for one training fold in Figure 5 and for multiple folds in Figure 6. While no ground truth is available, these results support the simulation studies' conclusions. Figure 5 illustrates the expected behavior of typical sparse methods in real-world settings: both the IS and JS estimates show many small, non-contiguous regions of selected voxels (although the JS estimates are somewhat smoother). More importantly, the IS and JS estimates suggest little to no joint support (in purple), which is clearly not consistent with findings from previous neuroimaging studies. In contrast, the SGL estimates show a substantial joint support attributable to the proposed sparse group penalty. Furthermore, this region is reasonably smooth, amenable to clinical interpretation, and stable across the (nearly identical) training folds (shown in Figure 6), unlike the IS and JS estimates. This conclusion regarding the stability of SGL estimates is supported in Table 3 by the higher Dice scores (both full and joint) between estimates obtained from different training folds. Numerically, all estimated models retain a comparable and moderate association between predicted and true age (shown by validation set MAE and *R*^2^ in Table 3).

**Table 3:** Performance results for the neuroimaging data analysis for 23-fold cross-validation.

**Method**	**MAE**		**Validation R^2^**	**Dice (pairwise)**	
	**Train**	**Validation**		**Full**	**Joint**
**IS**	**0.193 (0.051)**	**4.548 (1.563)**	**0.845 (0.171)**	0.529 (0.062)	0.395 (0.114)
**JS**	0.607 (0.188)	5.515 (1.640)	0.784 (0.183)	0.519 (0.070)	0.308 (0.131)
**SGL**	0.648 (0.117)	5.059 (1.967)	0.824 (0.150)	**0.678 (0.050)**	**0.666 (0.049)**

“*IS,” “JS,” and “SGL” refer to the independent sparse, joint sparse, and proposed sparse group lasso models, respectively. All results are presented in the form “mean (SD)”. Entries with the best mean results in each column are in bold*.

## 4. Discussion

### 4.1. Summary and Key Findings

Our simulation studies and analysis of a real-world neuroimaging dataset consistently supported our proposed SGL approach over both IS and JS models. In the simulation studies, SGL estimates of the full support gave notably higher (estimate-truth) Dice scores with lower variation across simulations. This result was strengthened when considering joint support detection and can be attributed to our inclusion of a sparse group lasso penalty. Figures 3, 4 illustrate the high false-positive rate and instability of IS and JS estimates and the difficulty they have in obtaining the joint support, especially for weaker signals. SGL estimates do not suffer in these ways and still recover the joint support even if the signal is weaker. A second set of simulation studies over independent datasets with the same ground truth similarly confirmed that our proposed method provides more stable estimates.

The neuroimaging analysis highlighted a major disadvantage of typical sparse methods (e.g., IS and JS), namely, their tendency to select a small number of voxels in non-contiguous regions, shown clearly in Figure 5. Furthermore, neither IS nor JS estimates suggested the presence of a joint support region, inconsistent with previous neuroimaging studies (Peters, 2002; Langkammer et al., 2012). These estimates again showed high instability, even with minimal variation in the training dataset during 23-fold cross validation (with total sample size *n* = 113). In contrast, our SGL method estimates a substantial joint support that was reasonably smooth, compact, and stable across training folds.

In both the simulation and neuroimaging analyses, all methods demonstrate a difference between the training and testing (or validation) set MAE, as expected. We attribute variation in this difference between the IS/JS and SGL models to the effect of overfitting when using the former methods, which is emphasized in the simulation studies (with about 82–90% of truly non-predictive voxels, much greater than the 32–50% estimated by the SGL model in the neuroimaging analysis). We suspect this variation is lessened in the neuroimaging analysis due to the naturally more complex data for which our simulation is a simple imitation.

### 4.2. Related Work and Impact

Existing approaches to multimodal neuroimaging data analysis commonly use techniques requiring a large sample size (Fan et al., 2007), analyze modes separately (Cherubini et al., 2009), aggregate voxel-wise measures to the structural level (Betts et al., 2016), or combine modalities in only an initial feature selection stage (Elkady et al., 2017). These approaches are either impractical or come at the cost of statistical power.

In this article, we proposed a unified, multimodal framework for joint region detection associated with a numerical variable of interest. Our work addresses the above limitations in existing neuroimaging analytic approaches as well as disadvantages of methods that employ sparse regularization. We are primarily interested in settings where overlap in the effect supports between modes is encouraged (rather than enforced) due to prior knowledge or a need for estimate interpretability. Our proposed method combines lasso, TV, and sparse group lasso penalties to this end.

Our primary motivating example concerns the association of R2* and QS MRI maps with age: in particular, previous works have shown that both modalities need to be considered simultaneously to delineate the complex association between iron and myelin levels (Hametner et al., 2018). An understanding of iron accumulation in healthy aging can give insights into the association between brain iron levels and neurological disease such as multiple sclerosis (Elkady et al., 2019). In this article, we considered the problem of identifying deep gray matter regions where variation in R2* and QS measures is jointly associated with aging in healthy controls. Although the main focus of this study was the detection of specific iron changes in deep gray matter, our work may be extended to delineate iron and myelin changes in myelin-rich white matter tissue.

The neuroimaging literature demonstrates a need for methods that can simultaneously accommodate multiple modalities and yield interpretable results in joint region detection problems, such as in our central R2*-QS-age example. Methods employing sparse regularization suffer due to spatial correlation in imaging data and tend to select a non-compact set of disjoint voxels. Image-based (e.g., TV) penalties help address this, but do not consider relationships between modalities. In practice, this may be problematic when it is expected that relevant brain regions are similar across modalities.

Our introduction of a sparse group lasso penalty is novel in this neuroimaging context and addresses the above gap in the current literature. We have shown that our SGL model is capable of obtaining estimates that are compact, interpretable, and stable, both within and between imaging modalities. Our work here is a first step in more general approaches to the multimodal analysis of age- or disease-related alterations in brain structure and function.

### 4.3. Limitations and Future Developments

In this article, we only considered a continuous response due to its wide applicability and our primary motivation to study age-related variation in DGM iron levels. However, our approach is readily applied to other tasks by modification of the objective function in (3). For example, Zhang et al. (2018) considers a discriminative anatomy detection problem based on logistic regression using a single MRI modality. This could be developed to include multiple modalities and a sparse group penalty for joint discriminative anatomy detection. To focus on our approach to multiple imaging modalities in this work, we also did not consider subject-level scalar predictors ***z***_*i*_ (e.g., demographic or clinical variables). By changing the ***y*** − *X*_1_***β***_1_ − *X*_2_***β***_2_ portion of the objective function in (3) to ***y*** − *Z**γ*** − *X*_1_***β***_1_ − *X*_2_***β***_2_, where *Z* has row vectors $z_{i}^{⊤}$, scalar covariates can be included with only straightforward changes to the ADMM algorithm. In both cases, our work here is a general framework that supports other standard tools employed in regression analysis.

## Data Availability Statement

The datasets presented in this study can be found in online repositories. The names of the repository/repositories and accession number(s) can be found at: https://github.com/pietrosa/qsr2-sim.

## Ethics Statement

The studies involving human participants were reviewed and approved by Health Research Ethics Board, University of Alberta. The patients/participants provided their written informed consent to participate in this study.

## Author Contributions

MP performed the analyses presented in this article, designed the simulation study, and was responsible for the text, tables, and figures in this work. LZ authored the optimization code used for model-fitting, provided some initial analytic neuroimaging results (not presented), and authored a previous version of this manuscript. PS was heavily involved in subject recruitment, acquired all neuroimaging data used in this study, and provided continued support with neuroimaging data handling and all pre-processing tasks. AE contributed to writing the introduction of the manuscript. AHW designed the image acquisition protocol, funded data collection, and led initial project discussions. LK provided methodological development, modified the general ADMM algorithm for this problem, and provided senior project supervision. DC provided methodological support and senior supervision, motivated this work, and contributed to previous versions of the manuscript. All authors contributed to manuscript editing.

## Conflict of Interest

The authors declare that the research was conducted in the absence of any commercial or financial relationships that could be construed as a potential conflict of interest.
